# Interference by vitamin B12-macrocomplexes: towards an effective detection and correct interpretation of hypo- and hypervitaminemia

**DOI:** 10.1515/almed-2024-0041

**Published:** 2024-07-12

**Authors:** José A. Delgado, María I. Pastor, Gemma Costa, Nuria Márquez, Josep Miquel Bauça

**Affiliations:** Department of Laboratory Medicine, Hospital Universitari Son Espases, Palma, Spain; Institut d’Investigació Sanitària de les Illes Balears, Palma de Mallorca, Spain

**Keywords:** interference, reference intervals, macromolecules, cobalamin, biomarker, B12

## Abstract

**Objectives:**

The presence of macro-B12 is a cause of factual hypervitaminemia B12. Precipitation with polyethylene glycol (PEG) is a simple method of removing high-molecular-weight biomolecules. However, “free” molecule may co-precipitate. This fact requires the establishment of reference intervals for the post-precipitation result. The main aims of the study were to: 1) establish post-PEG RIs for vitamin B12; 2) compare the two criteria for defining the presence of macro-B12; 3) evaluate the joint usefulness of percentage post-PEG vitamin B12 recovery and post-PEG RIs to inform the real vitamin B12 status; and 4), propose interpretative comments for ease of interpretation.

**Methods:**

Prospective study in which 488 serum samples from “healthy” individuals were included and analyzed to determine recovery and RIs following PEG precipitation. Subsequently, a retrospective analysis was carried out in order to evaluate the joint usefulness of both definitions for a suspicion of macro-B12. A total of 297 cases were included.

**Results:**

Recovery and post-PEG RIs determined on an Alinity i platform ranged from 60 to 107 % and from 118 to 506 pmol/L, respectively. McNemar’s test revealed statistically significant differences between both criteria for estimating interference. However, both methodologies showed good agreement. In 27 cases, the presence of macro-B12 coexisted with true hypervitaminemia B12. No case of coexistence of macro-B12 with vitamin B12 deficiency was detected in our series.

**Conclusions:**

Laboratory reports should include total vitamin B12 concentration, recovery and post-PEG vitamin B12 concentration with their adjusted RIs to better assess the body vitamin status.

## Introduction

Vitamin B12, also known as cobalamin, is an essential micronutrient that acts as a cofactor for the enzyme’s methionine synthase and methylmalonyl-CoA mutase, which are involved in DNA synthesis and intramolecular rearrangements, as well as in neurodevelopment [1].

Despite its limitations, the quantification of serum total vitamin B12 is the most widely used biomarker in clinical practice to assess vitamin B12 status and metabolism. A high number of requests are received in clinical laboratories on a daily basis, as it is an affordable and convenient test that is performed by immunoassay on automated and high-throughput analyzers. These requests are usually oriented in the quest for a deficiency, as the deficit of vitamin B12 is closely related to a number of chronic diseases and their associated problems, including neuropathy and cognitive disorders, anemia, cardiovascular disorders, age-related macular degeneration, and gastrointestinal diseases [2, 3]. However, a low vitamin B12 concentration in serum does not always imply a deficiency, just as a value within the reference range does not always connote normality [4, 5]. In this line, vitamin B12 tests based on immunoassays are not free from antibody-mediated interferences, including rheumatoid factor, heterophile antibodies, anti-intrinsic factor antibodies, and vitamin B12 macrocomplexes [6], [7], [8], [9]. Detection of such interferences by clinical laboratories is a true challenge, as they can mask the real assessment of the patient and lead to wrong medical actions. This is of special relevance in the case of vitamin B12, whose deficiency and excess are both associated with potentially severe pathologies, as reported elsewhere [5, 7, 10, 11].

The presence of vitamin B12 macrocomplexes (macro-B12), mostly due to immunoglobulin complexes, is a cause of factual hypervitaminemia B12 (hyper B12) that is little known by clinicians [5]. It consists of circulating high-molecular-weight molecules that are slowly cleared from the circulation and have neither biological activity nor any known clinical significance [7]. When a case of hyper B12 appears, early detection of possible macro-B12 interference by polyethylene glycol (PEG) precipitation helps the clinician to assess the need for further medical examinations, since it is possible to find cases of deficiency masked by interference, or the coexistence of interference with true hyper B12.

Screening for macro-B12 interferences is poorly established in routine laboratory practice, unlike for other biomarkers such as prolactin [12]. Further, there is no unanimous agreement regarding when to screen for macro-B12 in hyper B12 patients, neither is there a consensus on how to report post-PEG vitamin B12 concentration results. This lack of harmonization may lead to result misinterpretation.

Precipitation with PEG is a crude and simple method of removing high-molecular-weight biomolecules from a biological sample. However, it has the limitation that part of the “free” molecule may co-precipitate. This fact invalidates the use of the usual reference intervals for samples with the presence of such macromolecules, and imposes the need to establish new reference intervals for the post-precipitation result, given that the percentage recovery does not inform whether the serum concentration of the biomarker is within or outside normality, and therefore makes it impossible to evaluate the true vitamin B12 status of patients. Some authors, such as Solemaini et al. [13] obviate the use of percentage recovery as a definition of macro-B12 and define the presence of such interference when the absolute value of the total post-PEG vitamin B12 result is below the upper reference limit of the post-PEG reference range (including the 90 % confidence interval).

Based on these observations, as happens in the case of prolactin, it would be recommended the establishment of post-PEG reference intervals and their inclusion in laboratory reports together with the precipitation percentage, aiming for the correct interpretation of the status of the biomarker of interest [14].

Currently, in our hospital, a previously published protocol for the detection of antibody-mediated interferences in the total vitamin B12 assay is performed [15]. However, no post-PEG reference intervals are available to date.

By way of continuous improvement, and in accordance with the recommendations of scientific societies [15], this study had four objectives [1]: establish post-PEG reference intervals (RIs) for total vitamin B12 in our population by means of a prospective study [2]; compare and evaluate the concordance between the two bibliographic criteria for defining the presence or absence of macro-B12 by retrospective analyses [3]; evaluate the combined usefulness of percentage post-PEG vitamin B12 recovery and post-PEG RIs to inform the real vitamin B12 status (retrospective analyses), and, in this line; [4], propose interpretative comments for ease of interpretation.

## Materials and methods

These observational studies were performed at Hospital Universitari Son Espases (Palma de Mallorca, Spain), a tertiary level hospital that serves a direct population of 325,000 individuals and is a reference for a population of approximately 1,200,000 inhabitants. The population is predominantly Caucasian, with eating habits associated with the Mediterranean diet. The prospective study was conducted between October 2021 and June 2022, while the retrospective analysis included the period from July 2022 to June 2023. Analytical data were obtained from our laboratory information system GestLab (Indra, Spain), and clinical information was extracted from the Millennium hospital information system (Cerner Corporation, USA), after approval from the Ethics Committee of our institution [*Comité de Ética de la Investigación de las Islas Baleares* (CEI-IB), nº IB 4775/22 PI].

### Subjects

#### Prospective study: establishment of post-PEG RIs for total vitamin B12

##### Inclusion criteria

All individuals ≥15 years old whose results for the following magnitudes in a routine request were within their reference range were considered potentially includable: red blood cell count (men: 4.5–5.8×10^12^/L; women: 3.8–5.4×10^12^/L), hemoglobin (men: 130–167 g/L; women: 125–155 g/L), mean corpuscular volume (80–96 fL), white blood cell count (4.0–11.0×10^9^/L), platelet count (150–400×10^9^/L), alanine aminotransferase (ALT) (<55 U/L), total bilirubin (<20.5 µmoL/L), estimated glomerular filtration rate (CKD-EPI) (>90 mL/min/1.73 m^2^), protein in random urine (dipstick: <10^−1 ^g/L), folate (7.0–46.5 nmoL/L), vitamin B12 (140–554 pmoL/L), and urine methylmalonic acid (MMA) (0.52–5.75 mmol MMA/mol creatinine).

The reference intervals for both total vitamin B12 and urine methylmalonic acid were previously obtained in our laboratory through two prospective studies.

##### Exclusion criteria

The medical history of potentially eligible individuals was reviewed and those with the following were excluded: supplementation with vitamin B12 or folate; any cause leading to disorders in vitamin B12 metabolism such as alcoholism, liver disease, CKD, autoimmune diseases, infectious diseases (HIV, hepatitis), and hematological diseases; previous diagnosis of solid cancer or gastrointestinal disease (Crohn’s, malabsorption); genetic disorders such as methylmalonic aciduria; and individuals presenting symptoms of neuropathies (numbness and tingling in hands and feet, walking difficulty….) or cognitive impairment (dementia, Alzheimer’s disease…). In addition, individuals under treatment with proton pump inhibitors or metformin, pregnant women, as well as individuals whose serum total vitamin B12 results revealed antibody-mediated interferences as measured on the Alinity i platform (Abbott Diagnostics, USA) (vitamin B12 macrocomplexes, rheumatoid factor, and heterophile or anti-intrinsic factor antibodies) were also excluded. Those whose vitamin B12 recovery after precipitation with PEG was below 60 % of the original value were not included either. The cut-off points for confirmation or exclusion of interference were obtained in a previous study [15].

#### Retrospective study: usefulness of the criteria to define the presence or absence of macro-B12 separately and in combination

##### Inclusion criteria

**Figure 1:**
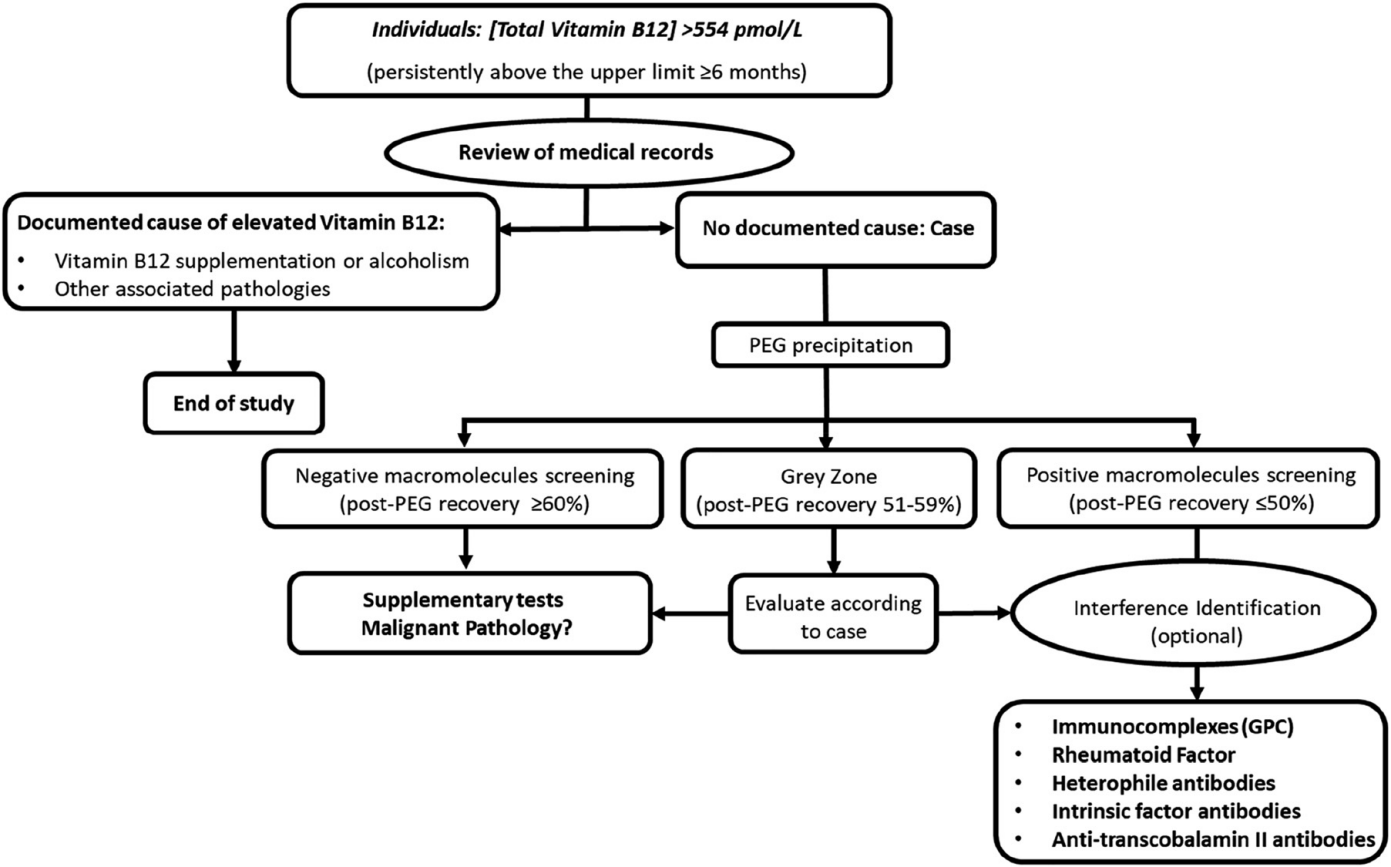
Algorithm for the screening of interferences due to vitamin B12 macrocomplexes in the clinical laboratoryen.

Individuals with hyper B12 (>554 pmoL/L) that met criteria for PEG precipitation according to the algorithm established in our laboratory (Figure 1).

##### Exclusion criteria

Individuals who previously showed confirmed interference in our vitamin B12 assay were excluded.

Regarding the second objective of our study, the diagnostic capacity for evaluation of macro-B12 using the definition of Solemaini et al. (based on the post-PEG vitamin B12 concentration) was compared to the classically used one (based on the percentage of vitamin B12 recovery).

In addition, in order to evaluate the combined usefulness of both criteria, the classification of cases was conducted based on the combined results for percentage recovery and their total post-PEG vitamin B12 result. It is considered the presence of antibody-mediated interference (mainly macro-B12) as *positive* when recovery was ≤50 %; as *negative* or *absent* when recovery was ≥60 %; and established a grey zone when recovery was between 51 and 59 %, where samples were considered *doubtful*. Regarding post-PEG vitamin B12 values, it is considered true hyper B12 status when the total post-PEG vitamin B12 value was above the upper limit for the reference range obtained and proposed by our laboratory, as well as true hypovitaminemia when the total post-PEG vitamin B12 value was below the lower limit.

### Analytical process

Vitamin B12 was measured in serum by a chemiluminescent immunoassay on the Alinity i platform (Abbott Diagnostics, USA). Measurement quality was ensured by the usual laboratory procedures, with a coefficient of variation (CV) below 6 % in the range of concentrations of the internal quality controls (269–674 pmol/L).

All serum samples from both studies underwent precipitation with PEG, following the classical protocol [16]: 200 µL of serum was mixed with 200 µL of a 25 % PEG-6000 solution. Samples were mixed and centrifuged at 2200 g for 15 min. Vitamin B12 concentration in the supernatant was measured. The percentage recovery of vitamin B12 after precipitation with PEG was calculated according to the following formula:

$$\text{Recovery }\left(\%\right)=\frac{2\;x\;\left[B_{12}\;\text{postPEG}\right]}{\left[B_{12}\;\text{prePEG}\right]}\;\times \;100$$

### Statistical analysis

The Kolmogorov-Smirnov test was used to assess the normality of the post-PEG vitamin B12 variable. Given the non-normality of the distribution, the reference interval was determined by non-parametric calculations (percentile 2.5–percentile 97.5). The McNemar test was used to compare proportions of related samples. Assessment of concordance between both definitions (percentage of post-PEG recovery vs. post-PEG total vitamin B12) to consider the presence or absence of macro-B12 was performed using Cohen’s Kappa index.

Statistical significance was established at 0.05.

Excel 2010 (Microsoft Inc, USA) and MedCalc v.19.3 (MedCalc Software Ltd, Belgium) were used for data processing.

## Results

For the prospective study, after applying the inclusion/exclusion criteria, a total of 488 individuals were included (217 men and 271 women, with a mean age of 47 years (interquartile range: 30–75), and a mean serum total vitamin B12 concentration of 328 pmol/L (interquartile range: 251–399). The post-PEG recovery ranged from 60 % (CI_95 %_: 59–61 %) to 107 % (CI_95 %_: 106–108 %), with a mean recovery of 90 %. As for the limits of the reference interval for total post-PEG vitamin B12 estimated from the 2.5th and 97.5th percentiles, the results were 118 pmol/L (CI_95 %_: 107–129) and 506 pmol/L (CI_95 %_: 494–517), respectively.

As for the retrospective analysis, a total of 297 cases met criteria for PEG precipitation according to the algorithm established in our laboratory. Considering the definition of macro-B12 based on the percentage of recovery (≤50 %), 105 cases (35 %) presented antibody-mediated interference, while 10 cases were reported as *doubtful* (3.4 %). However, if the criterion based on the total post-PEG vitamin B12 value proposed by Solemaini et al. had been applied, 102 cases (34 %) would have presented the aforementioned interference. Out of the 10 cases reported as *doubtful* according to the definition based on the post-PEG recovery, three of them would have been classified as macro-B12 according to Soilemaini’s criterion.

Regarding the statistical analysis, only the 287 cases reported as either *presence* or *absence* of macro-B12 interference were considered. The McNemar test revealed statistically significant differences (p-value <0.001) between both criteria for estimating interference. However, despite such statistical significance, both methodologies showed good agreement (corrected Kappa=0.65) in our study.

Table 1 shows the classification of the 297 cases that underwent PEG precipitation, considering the percentage recovery as a criterion for classification as presence/absence of macro-B12, and the total post-PEG vitamin B12 value as a criterion for true total vitamin B12 status. It is noteworthy that in 27 cases the presence of macro-B12 coexisted with true hyper B12: mean recovery 27 % (CI_95 %_: 24–31 %); mean total post-PEG vitamin B12 1,024 pmol/L (CI_95 %_: 776–1,270 pmol/L). No case of coexistence of the macro form with vitamin B12 deficiency was detected in our series.

**Table 1:** Retrospective analysis of cases undergoing PEG precipitation.

	Classification (% of recovery)		
	Absence of macro-B12 (≥60 %) n (%)	Doubtful macro-B12 (51–59 %) n (%)	Presence of macro-B12 (≤50 %) n (%)
Classification (post-PEG RI)		
Hypovitaminemia B12 (<118 pmol/L)	0	0	0
Normovitaminemia B12 (118 – 506 pmol/L)	21 (7.1 %)	3 (1.0 %)	78 (26.3 %)
Hypervitaminemia B12 (>506 pmol/L)	161 (54.2 %)	7 (2.3 %)	27 (9.1 %)

During the assessed period, 10 individuals underwent a second precipitation with PEG in an average timespan of 108 days. The average difference in the percentage of recovery between the second and first determination was 7 %. In no case did this difference lead to a change in the classification of the patient (presence or absence of macro-B12), that is, in seven of them the existence of hyper B12 without macro-B12 persisted, whereas in three the opposite situation occurred.

Interpretive comments to include on the laboratory report are shown in Table 2.

**Table 2:** Interpretative comments on the laboratory report for individuals with hypervitaminemia which underwent a PEG precipitation.

		Post-PEG percentage recovery			Comment
		Absence of macro-B12 (≥60 %)	Doubtful macro-B12 (51–59 %)	Presence of macro-B12 (≤50 %)	
Post-PEG vitamin B12 value	Hypovitaminmnemia B12 (<118 pmol/L)			X	Vitamin B12 deficiency after removal of interferences due to B12 macrocomplexes. We recommend further studies to evaluate functional B12 deficiency and vitamin supplementation.
	Normovitaminemia B12 (118 – 506 pmol/L)	X			Normal vitamin B12 concentration in the absence of B12 macrocomplexes. Normovitaminemia.
			X		Normal vitamin B12 concentration. The patient presented possible interferences due to vitamin B12 macrocomplexes (with neither biological activity nor clinical relevance) that were removed.
				X	Normal vitamin B12 concentration. The patient presented interferences due to vitamin B12 macrocomplexes (with neither biological activity nor clinical relevance) that were removed.
	Hypervitaminemia B12 (>506 pmol/L)	X			Hypervitaminemia B12 in the absence of interferences due to B12 macrocomplexes. In the absence of supplementation, we recommend further studies.
			X		Hypervitaminemia B12, confirmed after removal of possible interferences due to B12 macrocomplexes. In the absence of supplementation, we recommend further studies.
				X	Hypervitaminemia B12, confirmed after removal of interferences due to B12 macrocomplexes. In the absence of supplementation, we recommend further studies.

## Discussion

To the best of our knowledge, this is one of the few studies to establish a reference interval for total post-PEG vitamin B_12_ and perform an in-depth analysis of the usefulness of its inclusion in clinical laboratory reports.

With increasing laboratory automation, reduced cost, and wider accessibility of immunoassays, total vitamin B12 has become a common test performed in routine health checkups. Despite continuous improvements, antibody-mediated interferences have been reported for multiple analytical platforms [13, 15, 16]. PEG precipitation is an inexpensive, rapid, and simple method that is widely available for the detection of these interferences and is successfully applied in prolactin and thyrotropin assays [13], [14], [15], [16], [17], but scarcely implemented for total vitamin B12. Despite the advantages, PEG precipitation is not a specific method, and its interpretation should be performed with caution.

Traditionally, the percentage recovery of the biomarker has been used as an indicative point for the presence of interference by macroforms or antibodies that interfere with the immunoassay itself. However, this cut-off value lacks diagnostic specificity, given that the presence of interference may coexist with true hypo/hypervitaminemia B12 [7], as has been reported for other molecules such as prolactin [18, 19]. In these situations, the non-availability of a post-PEG reference interval may hamper the interpretation of results, and lead the clinician to misclassify patients. This fact has led scientific societies [14] to recommend that the concentration of the “free” form of the biomarker be included in the laboratory report together with appropriate reference intervals, if possible, established by the laboratory itself.

In our study, the results for both the range and mean percentage of post-PEG recovery agree with previous reported data [8, 13]. Nevertheless, regarding the reference intervals, although they are similar to those described by Solemaini et al. using the Cobas 8000^®^ platform [13] (122.1 pmol/L–514.4 pmol/L), they differ from those proposed by Öncel et al. [8]. These differences might be due to the use of a different platform and assay for the measurement of total vitamin B12 [Unicel DXI 800 (Beckman Coulter, USA)], a smaller sample size, different inclusion/exclusion criteria, and differences in the methodology for establishing RIs. Öncel et al. report a modification of the RIs proposed by the manufacturer based on the percentage of precipitation obtained in a control group. This methodology may not be advisable, as the values proposed by the manufacturers may not be well adjusted to the researchers’ population. As is the case of any analyte or biochemical magnitude, the establishment of post-PEG B12 RIs should always be performed according to CLSI recommendations [20].

As regards the comparison between methodologies for the definition of presence of macro-B12, the statistical analysis revealed significant differences between the two criteria. Notwithstanding, good agreement was found. Solemaini et al. [13] found a better approximation in the use of the total post-PEG vitamin B12 value, advocating the abandonment of the use of the percentage of recovery as a diagnostic criterion for the presence of macro-B12 interference. The main issue with the use of this methodology alone is the inability to discern true hyper B12 from the mixed situation (hyper B12 and macro-B12). From our perspective, the establishment of a post-PEG reference interval is an ideal complement to percentage of recovery, and should be included in laboratory reports, in line with SEQC-SEEN consensus guidelines for prolactin [14]. This conclusion is evidenced by the results from our retrospective analysis, where the separate use of either definition may be insufficient to assess a case in its entirety. The clear example is found in 27 of the cases (9.1 %) in which both macro-B12 and hyper B12 interference apparently coexisted (Table 1). Further, as shown in Table 1, more than half of the cases presented true hyper B12 without the presence of macro-B12 (54.2 %), which is similar to that reported by Öncel et al. [8] (55.2 %). Non-reporting of this hyper B12 finding to clinicians (through the laboratory report) may result in not performing further medical examinations that could clarify the reason for hyper B12.

Further, although in our retrospective analysis no cases of real hypovitaminemia were found during the assessed period, the use of post-PEG RIs would also enable its effective detection and reporting. Although this phenomenon is less common, its detection is equally essential in order to avoid the pathological effects caused by vitamin B12 deficiency.

In our study, it is found that 35 % of cases presented an antibody-mediated interference, which underlines the high prevalence of macro-B12 in selected patients with hypervitaminemia. A study of macro-B12 interference is rarely implemented in clinical laboratories, and medical staff has a great unawareness and inexperience of the subject alongside difficulties in interpreting results. Our group has tried to contribute to make this problem visible and has previously proposed a screening algorithm for macro-B12 interference that is simple, cost-effective, and easy to implement in clinical laboratories worldwide. In the present study, the intention is to go a step further and propose some recommendations on the laboratory report regarding the communication of macro-B12 interferences for individuals with hypervitaminemia which undergo a PEG precipitation, as outlined in Table 2:

– First, we advocate including in laboratory reports total vitamin B12 concentration, percentage of recovery, and post-PEG vitamin B12 concentration (*presumable free* B12) with their own adjusted RIs for the population served and referred to each laboratory’s total vitamin B12 assay.
– We also recommend accompanying the results of the report with explanatory or interpretative comments for clinicians for ease of interpretation and to be educational at the same time (Table 2).
– Finally, although further studies are required to investigate the persistence of macro-B12 forms; based on our experience and the information described in the literature regarding macroforms in other biomarkers [19], we consider that in patients with hyper B12 and previously ruled out macro-B12, it is unnecessary to repeat the precipitation procedure, thus alleviating the routine workload of endocrinology laboratories. In contrast, we do strongly recommend reporting the post-PEG B12 value (as concentration) in samples from patients with known interference, as this is crucial for proper follow-up of these patients.
  The main strength of our approach for RIs establishment is its prospective nature, as well as the sample size, compliance with CLSI recommendations for RIs establishment, and inclusion of multiple customized filters for the selection of healthy individuals. The review of medical records allows for the exclusion of individuals with a cause potentially affecting vitamin B12 metabolism.
  As for limitations, the application of a pseudo-direct methodology for RIs establishment relies on LIS and HIS records. Despite the careful and thorough selection of apparently healthy individuals, they are not actually true reference individuals, which also represents a shortcoming for the optimal establishment of reference intervals. As for the retrospective analysis, its very nature makes the comparison of both definitions for the presence of macro-B12 approximate due to the impossibility of confirming cases by chromatography.

## Conclusions

In cases where a PEG precipitation is performed, laboratory reports should include total vitamin B12 concentration, percentage of recovery, and post-PEG vitamin B12 concentration with their own adjusted RIs for the population served, in line with SEQC-SEEN consensus guidelines for prolactin, because the separate use of either definition may be insufficient to assess a case in its entirety, as shown in our study. This information (cases of hyper B12), together with explanatory comments in the laboratory reports, provides aims for the correct interpretation of the real status of this vitamin in the body and allows for early diagnosis and treatment, and therefore improves patient safety while reducing morbidity and mortality.
